# Microsphere-assisted hyperspectral imaging: super-resolution, non-destructive metrology for semiconductor devices

**DOI:** 10.1038/s41377-024-01469-3

**Published:** 2024-05-28

**Authors:** Jangryul Park, Youngsun Choi, Soonyang Kwon, Youngjun Lee, Jiwoong Kim, Jae-joon Kim, Jihye Lee, Jeongho Ahn, Hidong Kwak, Yusin Yang, Taeyong Jo, Myungjun Lee, Kwangrak Kim

**Affiliations:** 1 Metrology and Inspection Equipment R&D Team, Mechatronics Research, Samsung Electronics Co., Ltd., 1-1 Samsungjeonja-ro, hwaseong-si, Gyeonggi-do 18848 Republic of Korea; 2 Process Development Department, DRAM Process Development Team, Semiconductor R&D Center, Samsung Electronics Co., Ltd., 1-1 Samsungjeonja-ro, hwaseong-si, Gyeonggi-do 18848 Republic of Korea; 3 Process Development Department, Semiconductor R&D Center, Samsung Electronics Co., Ltd., 1-1 Samsungjeonja-ro, hwaseong-si, Gyeonggi-do 18848 Republic of Korea

**Keywords:** Super-resolution microscopy, Imaging and sensing

## Abstract

As semiconductor devices shrink and their manufacturing processes advance, accurately measuring in-cell critical dimensions (CD) becomes increasingly crucial. Traditional test element group (TEG) measurements are becoming inadequate for representing the fine, repetitive patterns in cell blocks. Conventional non-destructive metrology technologies like optical critical dimension (OCD) are limited due to their large spot diameter of approximately 25 μm, which impedes their efficacy for detailed in-cell structural analysis. Consequently, there is a pressing need for small-spot and non-destructive metrology methods. To address this limitation, we demonstrate a microsphere-assisted hyperspectral imaging (MAHSI) system, specifically designed for small spot optical metrology with super-resolution. Utilizing microsphere-assisted super-resolution imaging, this system achieves an optical resolution of 66 nm within a field of view of 5.6 μm × 5.6 μm. This approach effectively breaks the diffraction limit, significantly enhancing the magnification of the system. The MAHSI system incorporating hyperspectral imaging with a wavelength range of 400–790 nm, enables the capture of the reflection spectrum at each camera pixel. The achieved pixel resolution, which is equivalent to the measuring spot size, is 14.4 nm/pixel and the magnification is 450X. The MAHSI system enables measurement of local uniformity in critical areas like corners and edges of DRAM cell blocks, areas previously challenging to inspect with conventional OCD methods. To our knowledge, this approach represents the first global implementation of microsphere-assisted hyperspectral imaging to address the metrology challenges in complex 3D structures of semiconductor devices.

## Introduction

In the semiconductor manufacturing process, detecting and screening defects at an early stage is crucial for yield management^1–3^. Therefore, an appropriate metrology and inspection process for semiconductor device is essential. Spectroscopic analysis techniques have emerged as key tools in semiconductor metrology due to their non-destructive and rapid measurement abilities^4–6^. Spectroscopic ellipsometry (SE) and spectroscopic reflectometry are two representative spectroscopic techniques that are capable of measuring the thickness and critical dimensions (CD) of complex 3D structures with high sensitivity and precision^7–12^. Numerous attempts have been made to enhance their precision and sensitivity, such as expanding their spectral range to include ultraviolet or infrared, and utilizing advanced techniques like Mueller matrix or phase analysis^13–16^. Non-destructive metrology and inspection technologies for semiconductor devices face significant challenges as devices continue to decrease in size, increase in height, and become more complex^17,18^. The conventional use of SE to measure test element group (TEG) area, fabricated with dimensions larger than 40 μm × 40 μm, is limited by the need to reduce the illumination spot size for increasingly smaller target structures encountered in modern semiconductor devices^19–22^. The nano-dimensions of TEG and the requirements for measuring in-cell uniformity make it challenging for TEG to accurately represent the complex structure characteristics of current advanced devices^23–26^. This issue has led to the development of microellipsometry which aims to address these requirements. However, further improvements are necessary to achieve a smaller spot size of under 1 μm^27–29^

Recently, microsphere-assisted super-resolution imaging has emerged as a technique that enhances the magnification of optical system while overcoming the diffraction limit^30–34^. The photonic nanojet effect is one of the most representative theories for super-resolution enhancement by the microspheres, although its principles have not been fully elucidated^35–38^. The photonic nanojet refers to an energy concentration point on the shadow side of the microsphere, which is known to convert evanescent waves into propagating waves, resulting in super-resolution images within the virtual imaging plane. The microsphere used in this technique is typically made of transparent and dielectric materials, such as soda-lime glass, and has a radius ranging from 1 μm to 300 μm. Various attempts have been made to combine microsphere super-resolution and optical systems such as confocal microscopy and interferometry ^39–42^.

In our previous research^43^, we proposed microsphere-assisted spectroscopic reflectometry to reduce illumination spot size. This technique successfully reduced the diameter of the spectral measurement by 210 nm with a magnification of 530X. However, as the spot size decreases, there is a trade-off between resolution and throughput, which can be a potential concern since throughput is a crucial factor for expanding the measurement coverage or reducing turn-around time for semiconductor wafers. Moreover, as semiconductor device processes continue to advance, there is a growing need to measure in-cell uniformity, particularly in small areas and edge regions, which further increases the demand for a smaller spot size ^44,45^.

In this paper, we present the Microsphere-Assisted Hyperspectral Imaging (MAHSI) system, designed to drastically reduce the measurement spot size while maximizing throughput. The MAHSI system achieves a measurement spot size of 14.4 nm, equivalent to the system’s pixel resolution, and an optical resolution of 66 nm. To address chromatic aberrations and magnification changes induced by the microsphere, we implement magnification corrections for all hyperspectral images using image processing techniques. Additionally, we introduce an auto-focus and approach method to maintain non-contact conditions and safely and precisely approach the objective for in-line measurement, achieving a repeatability of 10 nm within 1 s. The advantage of the proposed system is experimentally validated by measuring standard film thickness samples and semiconductor devices. This system can address the current metrology challenges that cannot be solved by conventional SE with an illumination spot size.

## Results

### Microsphere-assisted hyperspectral imaging (MAHSI) system

**Fig. 1:**
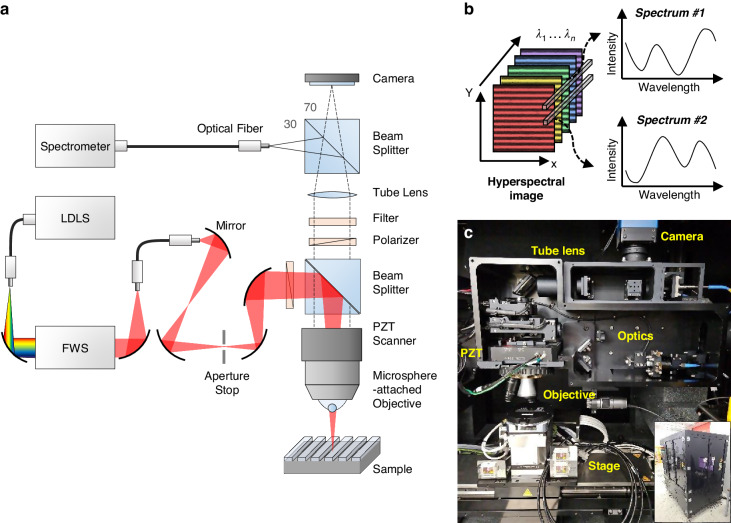
Configuration of the MAHSI system. **a** A schematic diagram of the MAHSI system based on a conventional microscopy. A Laser-Driven Light Source (LDLS) is used as the broadband light source. The Flexible Wavelength Selector (FWS) enables selection of incident light wavelengths, and the aperture stop adjusts the beam’s incident angle to the microsphere-attached objective lens. Reflected light from the sample is split and directed into both the camera and spectrometer. **b** The hyperspectral imaging process, the camera sequentially captures images with monochromatic wavelengths to obtain spectral reflectance at each pixel by interpolating intensity from each image. **c** Photos of the entire facility and internal optics and stage modules of the MAHSI system

This section describes the configuration of the MAHSI system and its utilization of hyperspectral imaging for semiconductor metrology applications. Figure 1a presents the MAHSI system, an advancement from the microsphere-assisted spectroscopic reflectometry system in our previous research^43^, incorporating a spectrometer as the detector. Key components of the system include a microsphere-attached objective lens and a flexible wavelength selector (FWS). The microsphere-attached objective lens is applied to enhance the magnification and break the diffraction limit. Stability and consistency are critical in semiconductor equipment systems, and the objective lens used in the MAHSI system serves as robust hardware, ensuring consistent measurement conditions and maintaining a non-contact status between the microsphere and the sample. The FWS with a monochromatic filtering unit in the MAHSI system has the capability to either transmit the incident light in a broadband range or filter specific wavelength ranges from 400 nm to 800 nm, with bandwidth variations from 3 nm to 10 nm. The aperture stop is used to adjust the incident angle to the pupil plane of the objective lens. The objective turret is set up with a piezo-electric scanner to control the vertical position of the objective lens precisely with an axial resolution of 1 nm. The beam reflected from the sample is directed through the objective and tube lenses, subsequently splitting into two beams for a spectrometer and a scientific complementary metal-oxide-semiconductor (sCMOS) camera. The spectrometer and camera acquire spectra for autofocus and hyperspectral images, respectively.

The MAHSI system operates by sequentially capturing hyperspectral images at different wavelengths to compile the spectral reflectance profile of the sample. Figure 1b illustrates the schematic of hyperspectral imaging. The camera of the MAHSI system captures multiple images at specific monochromatic wavelengths, filtered by the FWS. Subsequently, the spectrum in each pixel is obtained by interpolating the intensity values from the hyperspectral images corresponding to different wavelengths. Detailed descriptions of the hyperspectral imaging methodology and the components of the MAHSI system are provided in the Methods chapter.

A unique characteristic of the microsphere-attached objective lens used in the MAHSI system is its ultra-short working distance, which varies depending on the wavelength. Therefore, precise positioning of the microsphere and compensation of chromatic aberration are crucial in the MAHSI system. In the following section, we present a method for accurate positioning of the microsphere, enabling precise measurements of wavelength-dependent working distance.

### Microsphere scanning interferogram (MSI)

The working distance of the microsphere-attached objective lens is approximately 100 nm to 200 nm, depending on the operation wavelength^46^. Due to the extremely short working distance, there is a risk of contact between the lens and the sample, which can cause damage or contamination to the sample and the microsphere. Notably, the working distance is substantially less than the microsphere’s diameter, approximately 30 μm, resulting in a thin air gap between the microsphere and the sample. This gap forms a localized thin film around the optical axis, introducing interferometric characteristics due to multiple reflections.

**Fig. 2:**
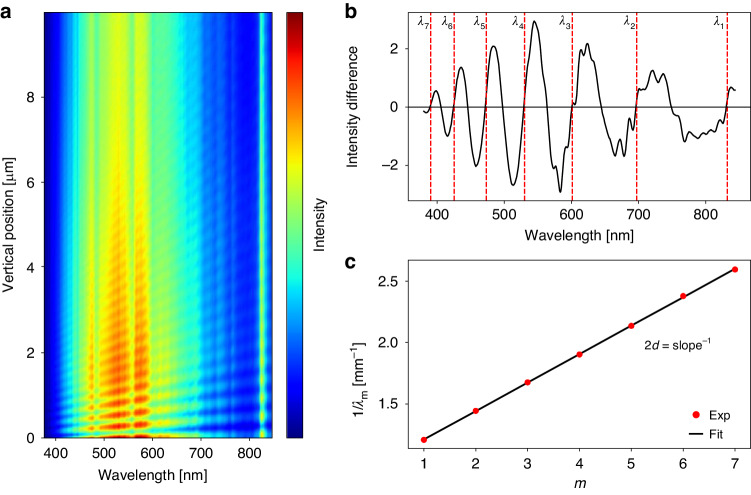
Measurement of distance between the objective lens and the sample. **a** Spectral intensities relative to the vertical position of the objective lens. **b** Interference nodes at a specific position, indicated by the spectrum difference between two adjacent positions of the microsphere-attached objective lens. **c** Linear fitting of the interference node data to determine the absolute distance between the microsphere and the sample

The presence of the thin film structure gives rise to interferometric characteristics due to multiple reflections, leading to oscillatory patterns in the measured reflection spectrum as the distance between the objective lens and the sample undergoes variations, as depicted in Fig. 2a. Consequently, it becomes necessary to incorporate the distance as a crucial parameter within the optical metrology model for the MAHSI system. To enable precise measurements of the distance and the analysis of interferometric reflection spectra, we develop the MSI method. In the proposed method, we sequentially measure two reflection spectra at two distinct vertical positions of the objective lens. This method allows for the estimation of system by fitting the measured spectra to the theoretical model described in Eq. (1)

1 $${f}_{{MSI}}\left(\lambda ,d,{x}_{1},\cdots ,{x}_{n}\right)=\frac{I\left(\lambda ,d,{x}_{1},\cdots ,{x}_{n}\right)}{I\left(\lambda ,d+\delta ,{x}_{1},\cdots ,{x}_{n}\right)}$$

where *λ* is operation wavelength, *d* is distance between the objective lens and the sample, *δ* is distance variation, and *x*_1_, …, *x*_*n*_ are system parameters such as film thickness and CDs. The proposed model function is based on the intensity ratio between two reflection spectra measured at different vertical positions of the objective lens, differing by a distance of *δ*. The theoretical calculation of this function is feasible because the spectral properties originating from the light source and optical components are effectively eliminated through self-normalization within the model. In the theoretical calculation, the air gap between the microsphere and the sample is treated as a thin film of constant thickness around the optical axis. The reflection spectrum *I*(*λ*, *d*, *x*_1_,…,*x*_*n*_) can be calculated using methods such as Fresnel equations and rigorous coupled-wave analysis for the simplified structure consisting of sample, air gap, and the microsphere. Direct fitting of the measured intensity ratio values to the model allows estimation of the lens-sample distance and other parameters without relying on additional reference spectrum measurements. The model fit is achieved by minimizing the difference between the measured *f*_MSI_ and the calculated *f*_MSI_, represented as Σ_*i*_ (*f* ^exp^_*i*_ − *f* ^theory^_*i*_)^2^.

This MSI method presents an advancement over conventional spectroscopic reflectometry, which typically requires a reference spectrum for reflectance calculation and system parameter estimation. By measuring two spectra within the sample, the MSI method independently estimates the working distance and other parameters, thereby mitigating the effects of temporal variations in the light source. This methodological enhancement is crucial for the precise metrology needed in advanced semiconductor manufacturing processes.

### MSI autofocus and distance measurement

For precise distance measurements and safe approach of the microsphere-attached objective lens, we employ the MSI method, which is modified specially for distance measurements. It is important to note that our system eschews the use of image-based autofocus and approach methods for the objective lens. Such image-based autofocus algorithms, which rely on real-time measurement of image sharpness, are unsuitable for the MAHSI system. This is due to the presence of multiple reflections between the microsphere and the sample, which can lead to several local optima, thus hindering the attainment of a globally optimal focus using conventional autofocus algorithms. Furthermore, for nanostructured samples beyond the optical resolution, accurate measurement of image sharpness becomes impractical.

The multiple reflections between the microsphere and the sample result in the interferometric feature shown in Fig. 2a. In the interference pattern, the spectral intensity varies periodically with distance, and this periodicity is dependent on the operation wavelength. The relation *mλ* = 2*d*, where *m* is the interference order and *λ* the operation wavelength, allows for distance verification through analysis of the interference pattern. It is noteworthy that the measurement in Fig. 2a was conducted on a silicon wafer with native oxide, but the method is broadly applicable to various samples, including semiconductor devices, as additional nanostructures on these samples minimally affect the primary interference pattern. As the distance increases, the oscillatory signal becomes weaker as shown in Fig. 2a, leading to errors in distance measurement. Overestimating the distance can cause the microsphere to make contact with the sample during scanner movement, based on the measured distance value. Therefore, the range of the distance measurement is limited to 10 μm. In the range below 10 μm, the validity of the proposed distance measurement is confirmed by the observed linearity between the encoder values of the scanner and the measured distance. The repeatability of the measurement is estimated to be 2.1 nm.

To measure the distance of the microsphere-attached objective lens at a specific position, reflection spectra are acquired by the spectrometer at two slightly separated vertical positions. The difference between these two spectra directly reveals the interference nodes, as illustrated in Fig. 2b. The distance, *d*, is determined by conducting a linear fit of the measured interference data represented by *λ*_*m*_, as shown in Fig. 2c. In the MAHSI system, the separation between the two positions is 10 nm, which depends on the desired resolution of the distance measurement. This resolution is limited by factors such as the signal-to-noise ratio of spectrometer and the repeatability of the scanner. Accurate measurement of distance enables precise positioning of the microsphere-attached objective lens, thereby ensuring the safety of both the microsphere and sample, and enhancing the repeatability and throughput of hyperspectral imaging. Once the working distance is measured, positioning of the microsphere-attached objective lens is accomplished without the need for additional vertical scanning.

### Chromatic aberration and magnification correction

In the process of microsphere imaging, focal positions at each wavelength vary due to chromatic aberration caused by the microsphere. This aberration leads to changes in magnification based on the optical characteristics of the microsphere. The magnified image generated by the microsphere is formed in a virtual image plane, dictated by the geometric relationship between the photonic nanojet of the microsphere and the sample^46,47^. The distance from the center of the microsphere to the photonic nanojet, *D*_*f*_, is changed by parameters such as the radius of the microsphere, *ϕ*_*μ*_, the refractive index of the microsphere, *n*_*μ*_, and the wavelength of the incident beam, *λ*_*i*_.

2 $${D}_{f}={f}_{1}\left({n}_{\mu },{\phi }_{\mu },{{\rm{\lambda }}}_{{\rm{i}}}\right)+{\varepsilon }_{1}({{\rm{\lambda }}}_{{\rm{i}}})$$

Note that the *ε*_1_ represents an error term resulting from the morphological imperfections of the microsphere and the non-ideal planar wave of the incident light through the microsphere. The distance between the sample and the magnified image by the microsphere, *D*_*v*_, corresponds to the focusing distance. This distance is determined by both *D*_*f*_ and the distance from the center of the microsphere to the surface of the sample, *D*_*s*_. The specific relationship between these distances is derived from the geometrical arrangement of the microsphere and the sample^43^. Therefore, the magnification is defined by the positional relationship between the distance *D*_*v*_, *D*_*s*_ and *D*_*f*_.

3 $${D}_{v}=\frac{{D}_{s}^{2}}{{D}_{f}-{D}_{s}}$$

4 $$M=\frac{{D}_{S}+{D}_{v}}{{D}_{S}}=\frac{{D}_{f}+{D}_{S}+{D}_{v}}{{D}_{f}}={f}_{2}\left({n}_{\mu },{\phi }_{\mu },{{\rm{\lambda }}}_{{\rm{i}}}\right)$$

Consequently, the focusing distance changes with varying wavelengths as *D*_*f*_ is a function of *λ*_*i*_, characterizing the chromatic aberration of the microsphere. Additionally, the magnification of the magnified image by the microsphere also varies depending on the wavelength. Therefore, for optimal focus, the working distance of the microsphere-attached objective lens is adjusted individually at each wavelength. Furthermore, the captured image at each wavelength are corrected to compensate for changes in magnification.

**Fig. 3:**
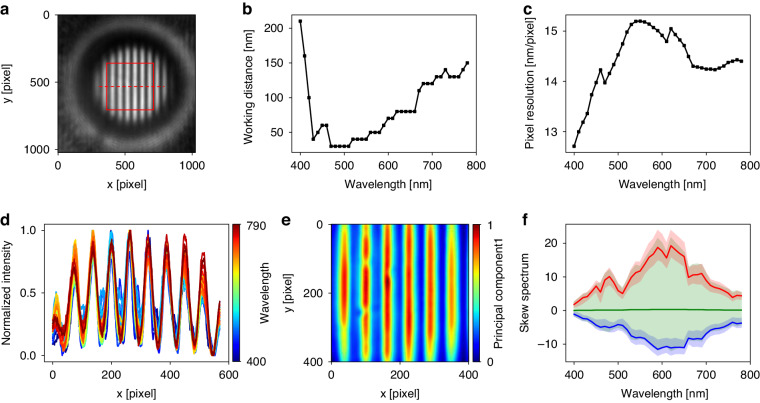
Correction of the magnification caused by chromatic aberration and PCA map. **a** The super-resolution image captured by the MAHSI system. The effective FOV is 5.6 μm × 5.6 μm size (red square). **b** Working distance, which is equal to focal position for each wavelength. **c** Pixel resolution for each wavelength. Averaged pixel resolution is 14.4 nm/pixel. Averaged magnification is 450X. **d** The result of magnification correction described by horizontal cut (red dot line). Aligned pixel data after normalization of magnification. **e**, **f** The PCA map and skew spectrum plot splits of line and space pattern. The red and blue colored regions are spectra of the top and bottom 10% of the principal component representing line and space pattern, respectively. The green colored area shows the remaining spectra representing line edge. The bold lines are average spectrum of the red and blue colored areas

To determine the working distance for each wavelength, a line and space pattern is imaged by the MAHSI system, as shown in Fig. 3a. The sample features a width of 0.5 μm and a pitch of 0.8 μm. Hyperspectral images are captured by sweeping the operation wavelength within a range from 400 nm to 790 nm at intervals of 10 nm. The hyperspectral imaging is repeated by scanning the distance between the objective lens and the sample, ranging from 20 nm to 1000 nm, in 10 nm steps. The focus score, based on the Sobel filter, is calculated from the captured images as a function of the vertical position to verify the position of the best focus^48^. The working distance for each operation wavelength is estimated, as shown in Fig. 3b. The working distance values measured by the MSI method allow for one-time access to the best focal position using the real-time measurement of the distance between the objective lens and the sample. By incorporating this approach, the system gains the advantage of enhancing the accuracy and reliability of the imaging results.

The captured hyperspectral images exhibit variations in magnifications as a result of chromatic aberration. The variation of magnification implies that the same pixel data at different wavelengths correspond to different sample locations. Therefore, a correction process is applied to all hyperspectral images to ensure accurate analysis and comparison by aligning them to a consistent magnification level. To verify the magnifications at each operation wavelength, the best focal images are analyzed. One-dimensional datasets, illustrated as red dashed line in Fig. 3a, are selected from the captured hyperspectral images to verify pixel resolution values at each operation wavelength.

These selected datasets are analyzed to obtain pitch values by applying a nonlinear curve fit with the model function (*ax*^2^ + *bx* + *c*)sin(2π(*x* – *x*_0_)/*p*). Here, *p* represents the pitch size in pixels, *x* denotes the pixel position, and the other variables represent the residual fitting coefficients. Considering the pitch size of the sample and the pixel size of the camera, pixel resolution values are calculated at each operation wavelength, as shown in Fig. 3c. A total of 40 hyperspectral images, adjusted to a consistent magnification level, are converted into a spectral dataset for each pixel. The results of magnification correction and normalized intensities in the horizontal profile are shown in Fig. 3d. This dataset generates a PCA map of the image as shown in Fig. 3e, where each pixel’s intensity corresponds to the principal component 1. The skew spectra clearly show their distinct splits between the line, space, and edge of the pattern as depicted in Fig. 3f, with differentiated regions marked in distinct colors. The average spectrum of each region is represented as a bold line line: blue line, space: red line, edge: green line, highlighting their unique characteristics.

The effective FOV with acceptable contrast deviation in image of the MAHSI system is 5.6 μm × 5.6 μm, which is represented by the red square in the Fig. 3a. The image contrast on the periphery of the microsphere is reduced, and the image is distorted caused by severe spherical aberration, with a contrast change of less than 5% in the effective. The camera sensor used in the MAHSI system has a pixel size of 6.5 μm × 6.5 μm, resulting in a pixel resolution of 14.4 nm/pixel. As a result, the effective magnification of the MAHSI system is calculated to be 450X, determined by the ratio of the pixel size to the pixel resolution.

### MSI model for 3D semiconductor device metrology

**Fig. 4:**
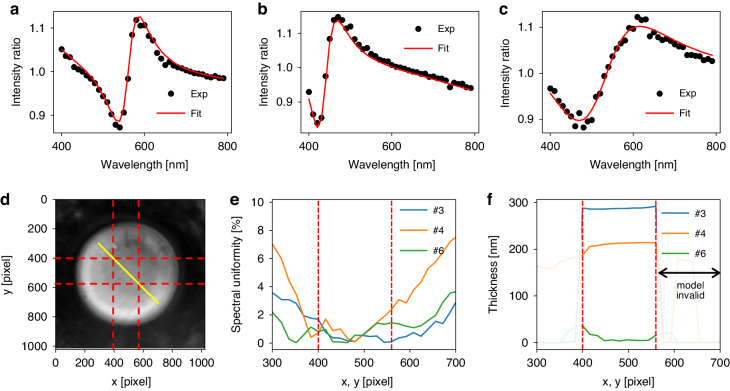
Film thickness measurements using the MSI model fit. Measured MSI spectra and their model fit for the three different SiO_2_ film samples (#3, #4, #6) where the desired film thickness values are 298.7 nm (**a**), 199.9 nm (**b**), and 3.5 nm (**c**), respectively. Spectral data is acquired with a 16 × 16 pixel binning at the center of captured images in (**a**–**c**). **d** Captured image of the sample. **e** Spectral uniformity along the line denoted in (**d**) as a solid yellow line. They are RMSE values between spectrum at the selected position and spectrum at the center. **f** Thickness values estimated by MSI fit. Dashed red lines in (**d**–**f**) represents the region where MSI model fit is valid

We demonstrated film thickness measurements using the MAHSI system to validate the performance of the MSI method, as shown in Fig. 4. Three different SiO_2_ film thickness samples are analyzed to estimate their film thickness values. Standard SiO_2_ samples are prepared and measured by RC-2 ellipsometer manufacutred by J.A. Woollam for the reference. More details about the samples and RC-2 are descrbed in section 4.4. At a constant working distance, a set of 40 images is acquired by sweeping the operation wavelength from 400 nm to 790 nm at 10 nm intervals to obtain reflection spectra across all horizontal positions in the field of view. This spectral acquisition is repeated while increasing the working distance by 10 nm to obtain MSI spectra calculated by Eq. (1). Throughout the measurement process, we maintain a working distance of less than 200 nm, a suitable value to satisfy the thin-film approximation requirements for the air gap while ensuring a non-contact condition between the microsphere and the sample. Using the MSI model fit, we estimate three parameters simultaeneously: the film thickness, the effective refractive index of the microsphere, and the working distance. The MSI model function is calculated using the transfer matrix method, employing a 4-layer model comprised of a microsphere, an air gap, a SiO_2_ film, and a Si substrate. The working distance in the model corresponds to the air gap’s thickness. The effective refractive index of the microsphere is introduced as a variable to adjust for numerical errors that may arise from the thin-film approximation of the air gap. The fitting results yield effective indices ranging from 1.2 to 1.7.

The spectra obtained at the center of the measured images are fitted with R-squared value greater than 0.975, as shown in Fig. 4a–c. We utilize 16 × 16 pixels binning during the spectral acquisition to reduce noise and compensate crosstalks. The fitting results yield estimated film thickness values of 292.1 nm, 201.0 nm, and 6.0 nm, respectively, compared to the RC-2 ellipsometer measurements of 298.7 nm, 199.9 nm, and 3.5 nm. The measurement results are tabulated in Table 1. The validity of the MSI model fit is confirmed within an area of approximately 2.5 µm × 2.5 µm, delineated by the red dashed lines in Fig. 4d, accounting for pixel resolution. The mesurement area of RC-2 is 200 µm × 500 µm which is much larger than MAHSI. Both MAHSI and RC-2 measurements are perforemd in the center of the square marker which is shown in Fig. 8b. Within this area, thickness distribution was successfully obtained, as shown in Fig. 4e, with R-squared values exceeding 0.95. As the measurement position of the spectrum deviates from the optical axis, the accuracy of the MSI model decreases due to the thin-film approximation constraints. Since the primary source of model inaccuracy arises from the thin film approximation, the previously mentioned magnification correction does not resolve the issue. Incorporating the curvature of the microsphere into the model may extend the region where the MSI remains valid, reducing the limitations imposed by the thin-film approximation and enhancing off-axis measurement accuracy.

**Table 1:** Comparison of the MAHSI and a conventional ellipsometer (RC-2, woollam)

Mesurement position	#3	#4	#6
MAHSI [nm]	292.1	201.0	6.0
RC-2 (Woollam) [nm]	298.7	199.9	3.5

Film thickness are measured at three different positions of a SiO_2_ standard film wafer (woollam) using the MAHSI system and RC-2 ellipsometer

### Device imaging and application

**Fig. 5:**
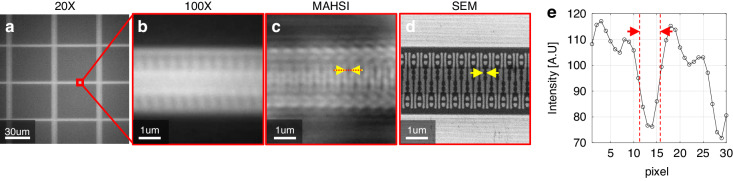
The SWD images of the DRAM device. **a** The microscope image with 20x of DRAM device. The cell and SWD area is shown. Images of the SWD area in DRAM imaged by x100 with 0.95 N.A. (**b**) and the MAHSI system (**c**) in ROI (red box). **d** The SEM image in same ROI. 66 nm CD indicated by yellow arrows. **e** The cross-sectional intensity profile of 66 nm CD indicated by red dot line in MAHSI image

Figure 5 presents images of the sub-word-line driver (SWD) area of a DRAM device captured using conventional microscopy, the MAHSI system, and SEM. Figure 5a depicts a conventional 20x microscopy image of a DRAM device. The SWD area in a DRAM device (red square), consisting of structures with CDs below 100 nm located between the cell blocks, cannot be resolved by conventional 100x microscopy due to the diffraction limit, as seen in Fig. 5b. Figure 5c, an MAHSI image captured at a wavelength of 410 nm, clearly resolves 66 nm CD lines, indicated by yellow arrows. Figure 5e show the cross-sectional intensity profile of 66 nm CD indicated by red dot line in MAHSI image. Figure 5d provides an SEM image for reference.

**Fig. 6:**
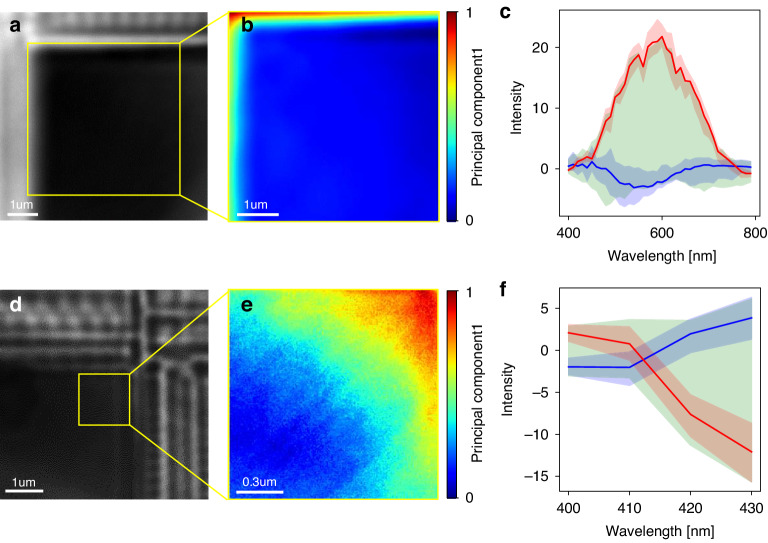
The spectral measurement result of two DRAM devices at different stages of manufacturing process imaged by the MAHSI system. **a** a monochromatic image of the DRAM cell block at 640 nm captured by the MAHSI system. **b** the PCA map by the MAHSI system in the peripheral area (yellow square) of the cell block. **c** the spectrum skew showing the average and deviation of the spectrum corresponding to the upper and lower 10% of principal component 1 value in blue and red, respectively, with the green region indicating other spectra. A monochromatic image (**d**), PCA map (**e**), and spectrum skew (**f**) captured by MAHSI of a DRAM cell block at a different processing stage contrasting with (**a**–**c**). **a**–**c** and (**d**–**f**) show different in-cell uniformity issues from two different processing stages of DRAM device

Figure 6 depicts a DRAM cell block image and its PCA map measured with the MAHSI system to observe in-cell uniformity. Figure 6a is a monochromatic image of the DRAM cell block at 640 nm captured by the MAHSI system, showing no variation in the corner and center area of the cell block. Figure 6b is the PCA map by the MAHSI system in the peripheral area (yellow square) of the cell block calculated from the spectral dataset within the wavelength range of 400 to 790 nm, where each pixel’s intensity corresponds to the principal component 1. At the center of the cell block denoted as Unit Block Center (UBC), represented by the blue region, principal component 1 values have little variation due to uniform repeating structures. However, at the edges of the cell block denoted as Unit Block Edge (UBE), depicted by blue and green regions, principal component 1 values change rapidly, indicating non-uniformity in the cell’s outer regions. In Fig. 6c, a clear spectrum separation is observed. This separation shows the average and deviation of the spectrum corresponding to the upper and lower 10% of principal component 1 value in blue and red, respectively, with the green region indicating other spectra. The spectra of the peripheral and central area of the cell are completely different in the 400–800 nm wavelength range, which means that the real structures are different. Figure 6d–f is a monochromatic image, PCA map, and spectrum skew captured by MAHSI of a DRAM cell block at a different stage of manufacturing process contrasting with Fig. 6a–c. The monochromatic image at 410 nm, shown in Fig. 6d, reveals no variation across the entire cell block area. However, imaging the edge and corner area (yellow square) of the cell block using the MAHSI system shows gradual changes in the principal component 1 values in the 400–450 nm wavelength range, as seen in Fig. 6e. This indicates more pronounced uniformity issues in the UBE than observed in Fig. 6a–c. Figure 6f demonstrates distinct spectrum separation, particularly highlighting changes at shorter wavelengths. The MAHSI system facilitates spectrum analysis within a wavelength range sensitive to structural variations.

As the semiconductor processes become more refined, the occurrence of such local structural uniformity issues is progressively more pronounced. Particularly, the uniformity issue around the periphery of the cell block and UBE are of concern in DRAM device manufacturing^49^. These structural uniformity issues could potentially impact the yield of modern DRAM device^50^. Therefore, as the importance of in-cell uniformity control increases, there is a need for non-destructive measurement of this region during the manufacturing process. The MAHSI technique enables the non-destructive metrology of these peripheral areas through hyperspectral imaging, while conventional OCD technologies are constrained by their limitations in spot size. Because the local change of a complex 3D structure of device causes a change in the spectrum, the MAHSI system could measure this in-cell locality variation of the 3D structure, especially the edge of the cell block.

## Discussion

In this study, we have demonstrated the capabilities of the MAHSI system, a pioneering technology enabling the acquisition of reflection spectra in a nanoscale spot with sub-diffraction limit optical resolution. This advancement is critical for measuring 3D structures in semiconductor devices. The MAHSI system, through hyperspectral imaging augmented by microsphere technology, achieves an optical resolution of 66 nm, breaking the conventional diffraction limit and enhancing pixel resolution. This methodology represents the first global instance of spectral measurement in a nanoscale spot using a microsphere-assisted hyperspectral imaging configuration.

A key innovation in our approach is the development of an accurate autofocus method tailored for the ultra-close working distances required by microsphere super-resolution optics. Due to the extremely short working distance around 100–200 nm in the MAHSI system, accurate autofocusing is crucial to avoid damage to the sample during imaging. We addressed this challenge by developing an autofocus method based on the interference signal generated by multiple reflections of incident light between the microsphere and the sample, referred to as MSI. This method accurately measures the distance between the microsphere and the sample with a repeatability and accuracy of under 10 nm in real-time.

This method is also utilized for accurate positioning at the best focus for each wavelength to compensate for the chromatic aberration of the microsphere. Because of the wavelength-dependent displacement of the photonic nanojet, chromatic aberration occurs, causing a change in the focal position for each wavelength. Therefore, best focal position is measured for each wavelength, and hyperspectral image are captured at each focal position using an accurate working distance measurement method. Additionally, because a change in the focal position results in a change in magnification, we measured the change in magnification in advance and applied magnification correction to each hyperspectral image. As a result, we have successfully performed the hyperspectral imaging with microsphere using the MAHSI system, achieving a magnification of 450X, and a pixel resolution of 14.4 nm/pixel, which indicates the measuring spot size of the spectral measurement. The MAHSI system captures hyperspectral images over a 5.6 µm × 5.6 µm FOV in 10.25 s. The collected hyperspectral images provide detailed spectral reflectance data for each pixel, which can be utilized for uniformity analysis through Principal Component Analysis (PCA) or measuring Critical Dimension (CD) values by fitting to the MSI model. This is a primary advantage of the MAHSI system that non-destructive metrology can be performed while simultaneously achieving super-resolution imaging.

In conclusion, the MAHSI system has been successfully utilized for monitoring the uniformity of the cell blocks in semiconductor devices, validating its feasibility for semiconductor device metrology. As semiconductor device processes become increasingly refined, the demand for spectral measurements to investigate nanostructure changes and uniformity in small areas escalates. In particular, as the need for direct measurements in the in-cell area increases instead of TEG, the importance of spectral measurements at in-cell areas with small spots has grown. The MAHSI system has successfully demonstrated the capability to detect changes in uniformity at the periphery of cell blocks through non-destructive spectral measurements with a 14.4 nm spot size. To our knowledge, this technique represents an innovative and effective solution for addressing metrology challenges in semiconductor device analysis.

## Methods

### The MAHSI system

The MAHSI system is based on spectroscopic reflectometry system with a microsphere-attached objective lens. The objective lens, a commercial product (SMAL by LIG Nanowise, U.K.), offers a magnification of 230X and a working distance ranging from 100 to 200 nm, with a depth of focus of approximately 200 nm. A white-light LDLS with an effective wavelength range from 370 to 920 nm is utilized as a light source. While the LDLS has a broader wavelength range extending from 170 to 2500 nm, its usage is limited due to the transmittance constraints of optical components and detectors. The system includes an optical fiber with a 100 μm core size, connected to a spectrometer covering a wavelength range of 200 to 1100 nm across 1044 pixels. The camera, featuring 2048 × 2048 pixels with each pixel measuring 6.5 μm × 6.5 μm, captures the reflected light signal from the sample at single wavelengths filtered through the FWS. The FWS facilitates wavelength sweep scans with a resolution of 1 nm within the range of 400 nm to 790 nm.

In order to perform hyperspectral imaging with microsphere-attached objective that has short working distance, a high-precision XYZ stage, and a piezo-electric scanner were used for stable stage control. For imaging a 300 mm wafer, the XY stage must have long travel ranges. The vacuum chuck for holding 300 mm wafers were setup up on the XYZ stage. The repeatability of the XY stage is 1 μm, and the repeatability of the Z stage is 0.2 μm. An objective turret was set up on the piezo-electric scanner with the resolution in axial direction of 1 nm. After course alignment of sample with XYZ stage, fine approach of the SMAL during the MSI autofocus and hyperspectral imaging was performed by using the piezo-electric scanner. This optics and system were set up on an active type vibration isolator controlled at −40 dB level, enabling precise control and preventing contact between SMAL and the sample.

### Theoretical model and fitting method of MSI

In the proposed system, reflection spectra depend on the distance between the microsphere and the sample due to the multiple reflection. Therefore, theoretical formulation of the reflection spectra includes the distance as a variable differently from a conventional spectroscopic reflectometry. In this case, normalization using reference spectra for eliminating spectral properties of light source is practically not applicable because the reference spectra are also sensitive to the distance. For this reason, we introduce microsphere scanning interferogram (MSI) for the model fit from which system parameters such as thickness and critical dimension (CD) are verified. MSI model fit is based on a ratio between two spectra taken from different vertical distance as shown in Eq. (1). Note that *f*_MSI_ approximately is 1 for a conventional SR where the reflection spectra do not depend on the distance within the depth of focus.

In the SiO_2_ film thickness measurement, the entire optical system of interest is a bilayer film structure composed of microsphere, air, SiO_2_, and Si where air gap between the microsphere and the sample is approximated as a thin film layer of constant thickness. Here, system parameters that can be verified by model fit are the air gap thickness which is the distance between the microsphere and sample, SiO_2_ thickness, and effective refractive index of microsphere. The effective refractive index of microsphere is introduced for compensation of residual errors caused from thin-film approximation. Consequently, the reflection spectra *I*(*λ*, *d*, *t*, *n*_S_) is theoretically obtained by Fresnel equation as a function of wavelength *λ*, distance *d*, film thickness *t*, and effective index of microsphere *n*_S_. The theoretical *f*_MSI_(*λ*, *d*, *t*, *n*_S_) also can be obtained by calculation of *I*(*λ*, *d*, *t*, *n*_S_) and *I*(*λ*, *d* + *δ*, *t*, *n*_S_) with the fixed distance difference *δ*.

For experimentally obtaining *f*_MSI_, two reflection spectra are measured at different distance *d* and *d* + *δ*. The distance difference *δ* should be much greater than the repeatability of the scanner, as the measurement error in *δ* can decrease the fitness of the model fit. Since the entire measurement of film thickness is based on thin-film approximation, the reflection spectra should be measured at the closest possible distance. Considering these conditions, the appropriate value of *δ* is determined as 10 nm, and the distance between the microsphere and the sample is maintained at around 100 nm. The theoretical model function *f*_MSI_(*λ*, *d*, *t*, *n*_S_) is fitted to the measured data to verify the film thickness *t*. In the model fit, we use sequential-least-squares-programming method. We note that the theoretical resolution of the thickness measurement is at a sub-nanometer level as a thickness change of 1 nm results in an RMSE of 5% in *f*_MSI_.

### The imaging sequence of MAHSI system

**Fig. 7:**
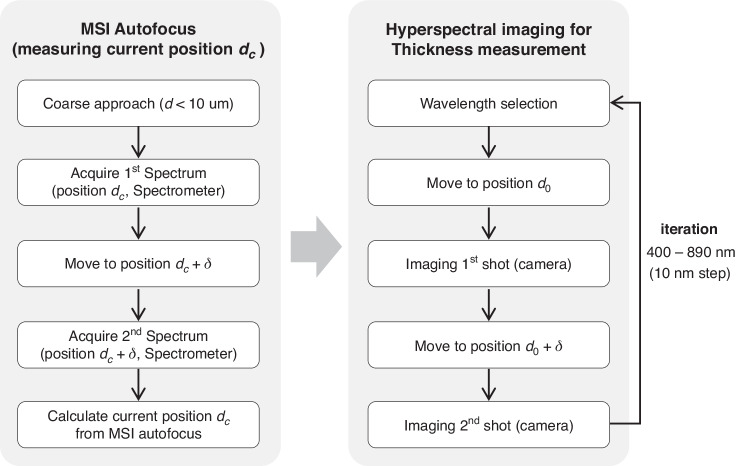
Imaging sequence of the MAHSI system. For acquiring hyperspectral imaging set, the MSI autofocus and the hyperspectral imaging are performed consecutively. *d* is distance between the objective and the sample and *δ* is distance variation. *d*_*c*_ is current distance between the objective and the sample. Images are obtained at positions *d*_0_ and *d*_0_ + *δ*, which is possible at any position where MSI is measured. The MSI autofocus including imaging and computation is performed within 0.1 s. And a total acquisition time of hyperspectral imaging that a total of 80 images are acquired within the 400 nm to 790 nm range at 10 nm steps is 19 s

Figure 7 shows the imaging sequence of the MAHSI system. For acquiring hyperspectral imaging set, the MSI autofocus and the hyperspectral imaging are performed consecutively. The MSI autofocus is the preparatory process to move objective to the focus position by measuring the current distance between the objective and the sample, *d*_*c*_. The *d*_*c*_ is calculated by the MSI model of the spectra obtained by spectrometer at two positions with a difference of 10 nm. The whole process of MSI autofocus including acquiring spectrum and calculation *d*_*c*_ is performed within 0.1 s.

After that, hyperspectral imaging is performed. In semiconductor applications, selecting the appropriate number of wavelengths for capture is crucial, considering the trade-off relationship between throughput and spectral resolution. In this demonstration, images are acquired at 40 wavelengths, a range of 400 nm to 790 nm with 10 nm steps. A bandwidth of 10 nm was used to ensure sufficient incident light power, despite the availability of bandwidth options at the FWS ranging from 3 nm to 10 nm for monochromatic light. Reference sample measurement confirms that there is no significant spectrum distortion with 10 nm bandwidth.

For thickness measurement by MSI model, two images are acquired according to each wavelength at the position *d*_*0*_ and *d*_*0*_ *+δ* to obtain MSI spectra calculated by Eq. (1). The position *d*_*0*_ and *δ* are fixed positions that does not change with wavelength. In this paper, *d*_*0*_ and *d*_*0*_ *+δ* are 100 nm and 110 nm above the sample. In this measurement, a total of 80 images were acquired within the 400 nm to 790 nm range at 10 nm steps. Each image is captured by the camera with an exposure time of 200 ms, ensuring sufficient of the incident light. FWS requires 50 ms to switch to another wavelength, and piezo-electric scanner requires under 10 ms to move. Therefore, total acquisition time is 19 s for capturing a hyperspectral imaging set of one FOV.

As shown in Fig. 6, only the intensity of the reflected spectrum is needed with MAHSI for locality analysis. A single set of hyperspectral data is only required since there is no need to use the MSI model. Therefore, a total of 40 images are acquired, resulting in a total acquisition time of 10.25 s. In this case, images are not acquired at the fixed position, *d*_*0*_, for all wavelengths, but at the focal position for each wavelength. After the wavelength is selected, only one image is acquired at the focal position, which retrieves a library that is pre-measured focal position at each wavelength. And, this process is repeated for each wavelength, resulting in a total of 40 images being acquired.

If the microsphere-attached objective is unchanged, the focal position at each wavelength does not change. This is measured when the microsphere-attached objective lens is first set up. The focal position is determined by finding the point where the sharpness is maximized from scanning images of sufficient distance in z-direction for each wavelength. The focal position at each wavelength of the microsphere-attached objective used in this paper is shown in Fig. 3.

### Film thickness measurement by the conventional ellipsometer

Ellipsometry is a widely used technique for measuring thin film thickness and material properties. In this paper, we utilize the RC-2 ellipsometer from J.A. Woollam to compare film thickness measurements with those obtained using the MAHSI method. The RC-2 has a wavelength range of 210 to 2500 nm and a measurement area of 200 μm × 500 μm. It is capable of performing automatic measurements on 4-inch wafers, providing a thickness map of the entire wafer.

**Fig. 8:**
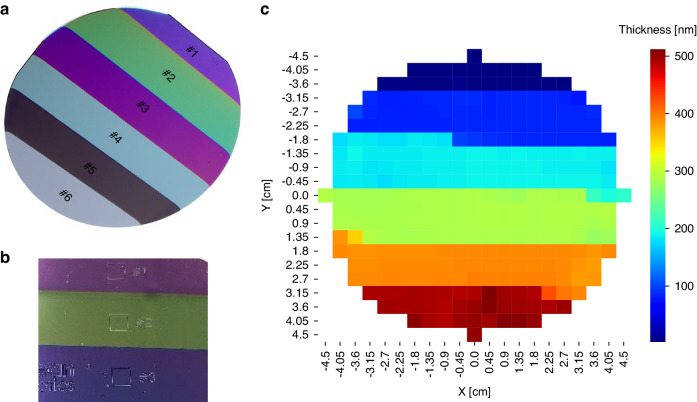
Standard SiO_2_ sample and thickness measurement map. **a** SiO_2_ film over Si wafer with 6 different thickness labeled from #1 to #6, and thickness specifications from manufacturer are 494.4 nm, 392.1 nm, 288.3 nm, 187.2 nm, 83.1 nm and 3 nm. **b** image of the square box which is the guaranteed area by the manufacturer. **c** Full wafer thickness map measured by RC-2 ellipsometer

The standard film sample used in this study consists of a SiO_2_ film layer deposited on a 4-inch silicon wafer. The sample features six different film thicknesses, as shown in Fig. 8a. Each region is marked with a square box, as depicted in Fig. 8b, where the manufacturer guarantees the thickness of the film. The thickness values from #1 to #6, as specified in the manufacturer’s sheet, are 494.4 nm, 392.1 nm, 288.3 nm, 187.2 nm, 83.1 nm, and 3 nm, respectively.

To ensure accurate measurements, we performed ellipsometric measurements in the target squares for #3, #4, and #6 using RC-2. This was done to account for potential oxidation or contamination of the wafer. The results are presented in Table 1. Additionally, the full thickness map of the standard wafer obtained using RC-2 is depicted in Fig. 8c. The measurement pitch is 0.45 cm in both the x and y directions, with a total of 316 measurement points.
